# Do acute changes in ambient air pollution increase the risk of potentially fatal cardiac arrhythmias in patients with implantable cardioverter defibrillators?

**DOI:** 10.1186/s12940-020-00622-w

**Published:** 2020-06-18

**Authors:** Robert Dales, Douglas S. Lee, Xuesong Wang, Sabit Cakmak, Mieczyslaw Szyszkowicz, Robin Shutt, David Birnie

**Affiliations:** 1 Environmental Health Science and Research Bureau, Health Canada, and Ottawa Hospital Research Institute, University of Ottawa, 101 Tunney’s Pasture Driveway, Ottawa, ON K1A 0K9 Canada; 2 ICES, Peter Munk Cardiac Centre of University Health Network, University of Toronto, Toronto, Canada; 3 ICES, Toronto, Ontario Canada; 4 Environmental Health Science and Research Bureau, Health Canada, Ottawa, Canada; 5 Arrhythmia Service, Department of Medicine, Heart Institute, University of Ottawa, Ottawa, Canada

**Keywords:** Air pollution, Cardiac rhythm, Epidemiology, Implantable cardioverter defibrillator

## Abstract

**Background:**

Daily changes in ambient air pollution have been associated with cardiac morbidity and mortality. Precipitating a cardiac arrhythmia in susceptible individuals may be one mechanism. We investigated the influence of daily changes in air pollution in the Province of Ontario, Canada on the frequency of discharges from implantable cardio defibrillators (ICDs) which occur in response to potentially life threatening arrhythmias.

**Methods:**

Using a case- crossover design, we compared ambient air pollution concentrations on the day of an ICD discharge to other days in the same month and year in 1952 patients. We adjusted for weather, lagged the exposure data from 0 to 3 days, and stratified the results by several patient-related characteristics.

**Results:**

Median (interquartile range) for ozone (O_3_), fine particulate matter (PM_2.5_), sulphur dioxide (SO_2_) and nitrogen dioxide (NO_2_) were 26.0 ppb (19.4, 33.0), 6.6 μg/m^3^ (4.3, 10.6), 1.00 ppb (0.4,2.1), 10.0 ppb (6.0,15.3) respectively. Unlagged odds ratios (95%) for an ICD discharge associated with an interquartile range increase in pollutant were 0.97 (0.86, 1.09) for O_3_, 0.99 (0.92, 1.06) for PM_2.5_, 0.97 (0.91, 1.03) for SO_2_, and 1.00 (0.89, 1.12) for NO_2_.

**Conclusion:**

We found no evidence that the concentrations of ambient air pollution observed in our study were a risk factor for potentially fatal cardiac arrhythmias in patients with ICDs.

## Background

Administrative database studies have linked daily changes in ambient air quality with an increased risk of hospitalization and death from cardiac disease [1, 2]. To what extent this association is due to dysrhythmia, ischemia, heart failure, or a combination of these diseases is unclear [3]. There is evidence that cardiac arrhythmia may be playing a role because it is thought to be a common cause of sudden cardiac death in a community setting [4], and air pollution can modify cardiac autonomic tone and thereby change cardiac rhythm [5]. Finding an association between daily levels of air pollution and the frequency of potentially life-threatening cardiac arrhythmias would provide additional evidence to support this hypothesis and strengthen the evidence for causality.

An implantable cardioverter-defibrillator (ICD) is an electrical impulse generator implanted in patients who are at risk of sudden cardiac death due to ventricular fibrillation/tachycardia. A potentially fatal arrhythmia triggers overdrive pacing or defibrillation to restore the baseline rhythm. A systematic review and meta-analysis of seven studies investigating the association between ventricular arrhythmias and air pollution in patients with ICDs reported inconsistent results. For the majority of the studies the lower confidence interval of the odds ratios (OR) did not exceed one for carbon monoxide (CO), nitrogen dioxide (NO_2_), ozone (O_3_), sulfur dioxide (SO_2_), particulate matter < 2.5 μm in diameter (PM_2.5_), and particulate matter < 10 μm in diameter (PM_10_) [6]. One study of 281 subjects in Italy reported a concentration-response function whereby PM_2.5_ concentrations greater than 25 μg/m^3^ were associated with a non-linear increase in the risk ratio for ventricular tachyarrhythmias. No effect was seen for PM_10_ or gaseous air pollutants [7].

The Ontario Ministry of Health, the sole payer of ICDs in Ontario, Canada had mandated that all ICD recipients have their data entered into the Ontario ICD database [8]. We used this large database to investigate the influence of ambient air pollution on the frequency of appropriate ICD discharges.

Ontario has the largest population of any Canadian province with more than a third of all Canadians. The ambient air pollution is generally within the Canadian Ambient Air Quality Standards (CAAQS) recommended guidelines [9]. The purpose of the present study is to investigate whether air pollution, which meets current concentration standards, triggers ICD discharges. Examining the evidence for an association between daily levels of air pollution and cardiac rhythm disturbances will enhance understanding of the health risks of air pollution in a susceptible population, the physiologic mechanisms underlying the cardiac toxicity of air pollution, and the nature of the association between cardiac morbidity and air pollution at lower concentrations than have been observed in the past.

This study addresses several knowledge gaps. Little is known about the effects of low concentrations of air pollution on ICD discharges. Though concentrations of nitrogen dioxide (NO_2_) and fine particulate matter (PM_2.5_) have been decreasing for decades in both the United States and Canada [10, 11], recent studies have found adverse effects of air pollution at concentrations within existing air quality standards [12]. Information about the lower thresholds for adverse health effects is essential to making informed decisions about air pollution regulation. Previous Canadian research in this area was done 16 years ago on patients with ICDs [13]. No significant association was found but the sample size was small compared to the sample size of the current study. The large sample size in the present study allowed us to stratify results by geographic region and several clinical variables in order to look for potentially susceptible subgroups.

## Methods

### The Ontario ICD registry

This database is kept by the Institute of Clinical Evaluative Sciences (ICES) in Toronto. Between two and three thousand defibrillators were implanted each year in residents of the Province of Ontario, between January 2007 and 2012. Patients were followed until death or their first appropriate ICD discharge, defined as being in response to ventricular fibrillation or ventricular tachycardia independently verified by a cardiologist [8]. The last recorded ICD discharge was May 2012, and survivors were followed until as late as May 31, 2012. There were no exclusions based on age, geographic location or treating medical facility. Patients were eligible if they had had an ICD implant for primary or secondary prevention, or if the ICD was replaced. If during the study period, an ICD change was required, then we kept the data only for the first ICD. The study was approved by Health Canada and Public Health Agency of Canada’s Research Ethics Board. Study Design: We used a case crossover design restricted to the patients within the ICD registry who had an appropriate ICD discharge. We compared the average concentration of air pollution on the day of an ICD discharge (case day) with the mean concentration measured on every other same day of the week (i.e. 7 days apart), in the same month and in the same year of the discharge (control days). For example, if a discharge occurred on a Sunday in September 2009, the control periods would be every Sunday in that month and year, apart from the day of discharge. Each patient served as their own control and by design, day-of-the week, month and year are also controlled for as well as short term time-invariant characteristics such as age, sex, social status, place of residence and chronic health conditions.

### Air quality data

Ambient air pollution data were provided by the National Air Pollution Surveillance System (NAPS), Environment Canada [14]. Data are collected hourly, 24 h a day, every day. Daily mean temperature, barometric pressure and relative humidity were obtained from the National Climate Data and Information Archive [15]. To estimate personal exposure, we used data from the NAPS and weather monitors closest to each subject’s area of residence, identified by a three digit postal code. Patients who did not reside within 50 km of a monitor (which is estimated to be less than 10%) were excluded from the study. Those who lived within 50 km of only one monitor were assigned the values measured at that single site. Those who lived within 50 km of more than one monitor were assigned exposure estimates based on the average of these stations, weighted inversely by the distances between the residential neighborhood and each of the monitors. Of the 516 residential areas indicated by the 3 digit postal code in Ontario, 482 were within 50 km of at least one NAPS monitor. In addition to the individual daily mean concentrations of sulphur dioxide (SO_2_), ozone (O_3_), nitrogen dioxide (NO_2_), and fine particulate matter with aerodynamic diameter ≤ 2.5 μm (PM_2.5_), we used the Canadian Air Quality Health Index (AQHI) which is a summary measure of the latter three individual pollutants [16].

It was not feasible to have exact personal exposure measures. We assumed that on days when NAPS monitors recorded higher values, personal exposure was likely to be higher than when NAPS reported lower concentrations. The analysis is based on the difference in air pollution between event and control days, not absolute levels of exposure.

### Statistical analysis

Within the ICD database t-tests were used to compare clinical characteristics between those who experienced an ICD discharge and those who didn’t. Cox proportional hazards regression analyses provided relative risk estimates for calculating hazard ratios. For the case-crossover analysis, which was restricted to only those who experienced an ICD discharge, conditional logistic regression was employed using SAS software, SAS Enterprise Guide, Version 7.1 Copyright (2015) SAS Institute Inc., Cary, North Carolina, USA. Daily mean temperature, relative humidity and barometric pressure were included in the models as continuous independent variables. Restricted cubic spline terms for weather variables fit using SAS macro %daspline (DSHIDE). Five knots were chosen, at 5th, 27.5th, 50th, 72.5th and 95th percentiles [17, 18]. We tested the possibility of delayed effects by using lags of 0,1,2,3 days between air pollution and ICD discharge. To assess the effect of cumulative exposure, the mean of air pollution averaged over the day of the discharge and the 3 previous days was also tested for an association with ICD discharge. Goodness of fit was tested using Akaike Information Criterion [19]. To determine if there were susceptible subgroups within the study population, we stratified results by univariate predictors of ICD discharge. To assess the possibility that an effect of air pollution on discharges may be affected by small differences in long term mean air quality or weather, we stratified results by region.

## Results

### Air pollution concentrations

Sixty NAPS were used for this study. All measured O_3_, 54 measured PM_2.5_, 45 measured NO_2_ and 37 measured SO_2._ Mean daily values of air pollutants were relatively low (Table 1), within the acceptable ranges according to the American and Canadian National Standards [10, 20]. The observed 24 h mean for PM_2.5_ was 8.2 μg/m^3^ standard deviation (SD) 5.6. The US standard is a 98 percentile value of 35 μg/m^3^ averaged over a 3 year period, and the Canadian 2015 standard was 28 μg/m^3^. The observed mean daily AQHI value of 2.7 (SD 0.75), on a scale of 1 to 10, would be considered in the lower range of health risk [21]. Though Ontario is a large province (over 1 million square kilometers), the majority of patients experienced a similar moderate continental climate. The 24 h mean temperature was − 0.07 °C (SD 7.7) in the cold season from October to March and 15.7 °C (SD 6.1) in the warm season from April to September.

**Table 1:** Daily mean, median, standard deviation (SD) and interquartile ranges (IQR) for air pollution and weather variables during the period 2007 to 2012

	Overall			Warm		Cold	
	Days of observation * 10^5 *^	Mean (SD)	Median (IQR)	Mean (SD)	Median (IQR)	Mean (SD)	Median (IQR)
**Weather**							
Temperature, °C	9.81	7.85 (10.53)	8.49 (−0.28,17.07)	15.74 (6.12)	16.87 (11.84,20.46)	−0.07 (7.70)	−0.10 (−5.52,5.23)
Relative humidity, %	9.65	73.19 (12.27)	73.95 (65.50,82.08)	70.65 (12.70)	71.45 (62.33,79.89)	75.75 (11.26)	76.21 (68.56,83.96)
Barometric pressure, kPa	9.62	99.32 (1.19)	99.45 (98.54,100.17)	99.23 (1.12)	99.39 (98.51,100.04)	99.41 (1.26)	99.53 (98.58,100.33)
**Air Pollutant**							
SO_2_, ppb	7.14	1.90 (3.06)	1.00 (0.43,2.14)	1.72 (3.07)	1.00 (0.24,2.00)	2.09 (3.04)	1.15 (0.61,2.82)
NO_2_, ppb	8.74	11.44 (6.78)	10.04 (6.02,15.31)	9.43 (5.60)	8.16 (5.01,12.57)	13.46 (7.24)	12.37 (8.00,18.00)
Ozone, ppb	9.23	26.52 (9.71)	26.00 (19.44,33.00)	30.37 (9.55)	30.00 (23.37,36.61)	22.64 (8.20)	22.33 (16.63,28.59)
PM_2.5,_ μg/m^3^	9.19	8.21 (5.60)	6.57 (4.26,10.55)	8.47 (5.76)	6.91 (4.29,10.84)	7.95 (5.42)	6.32 (4.19,10.52)
AQHI	8.58	2.70 (0.75)	2.63 (2.19,3.13)	2.72 (0.80)	2.66 (2.15,3.21)	2.68 (0.70)	2.61 (2.21,3.06)

^*^Number of days of observation for each forward 3-digit postal code area multiplied by the number of these areas in the study

### Description of patients in the ICD database

Data were collected data on just over ten thousand patients, mostly men, between 2007 and 2012. Follow-up during that time period ceased if there was an ICD discharge or death (Table 2). The average length of follow-up was 2.2 years with a maximum of 5.3 years. A subset of these patients has been previously described [22]. Of the 19 % (1952) who registered an appropriate ICD discharge, primary prevention was the indication for an ICD in 42% of the cases. Sixty-six percent had ischemic heart disease, 35% had a history of atrial fibrillation, 47% had sustained ventricular tachycardia, and 55% had a severely reduced ejection fraction. Apart from ischemic heart disease and cardiomyopathy, other cardiac pathologies included valvular and hypertensive heart diseases. Greater than 80% of patients who experienced an ICD discharge had been prescribed a beta-blocker and either an angiotensin-converting enzyme (ACE) inhibitor or an angiotensin II receptor blocker (ARB).

**Table 2:** Characteristics of patients in the ICD database stratified by the occurrence of an appropriate ICD discharge. Results are expressed as mean ± standard deviation or n (%)

	Appropriate ICD discharge		***P***-value (2-sided)
	No	Yes	
	*N* = 8368	*N* = 1952	
Age (year)	64.43 ± 12.98	65.28 ± 12.37	0.009
Male	6510 (77.8%)	1665 (85.3%)	< 0.001
Current cigarette smokers	1126 (13.5%)	277 (14.2%)	0.02
Implant indication			
Primary prevention	4586 (54.8%)	827 (42.4%)	< 0.001
Secondary prevention	1749 (20.9%)	661 (33.9%)	
Replacement	2033 (24.3%)	464 (23.8%)	
Primary heart disease			
Ischemic	5380 (64.3%)	1282 (65.7%)	< 0.001
Dilated cardiomyopathy	1821 (21.8%)	460 (23.6%)	
Other	1167 (13.9%)	210 (10.8%)	
Atrial fibrillation	2677 (32.0%)	681 (34.9%)	0.014
Documented ventricular arrhythmia			
Sustained tachycardia, Fibrillation or arrest	2723 (32.5%)	910 (46.6%)	< 0.001
Non-sustained tachycardia	992 (11.9%)	311 (15.9%)	
None	4653 (55.6%)	731 (37.4%)	
Left ventricular ejection fraction			
≤ 30%	4605 (55.0%)	1068 (54.7%)	0.91
31–40%	1498 (17.9%)	362 (18.5%)	
> 40%	1456 (17.4%)	339 (17.4%)	
Missing	809 (9.7%)	183 (9.4%)	
Medications			
β-adrenoreceptor antagonist	7204 (86.1%)	1695 (86.8%)	0.39
ACE inhibitor or ARB^a^	6722 (80.3%)	1632 (83.6%)	< 0.001
Spironolactone	2120 (25.3%)	520 (26.6%)	0.23
Loop diuretics	4432 (53.0%)	1055 (54.0%)	0.39
Digoxin	1713 (20.5%)	456 (23.4%)	0.005
Amiodarone	1543 (18.4%)	443 (22.7%)	< 0.001

^a^Angiotensin-converting enzyme inhibitor or angiotensin II receptor blocker

Statistically significant hazard ratios for the univariate predictors of an appropriate ICD discharge (*p* < 0.05) were: older age, male sex, history of smoking, ICD placement for secondary prevention, atrial fibrillation, and previously documented VT (ventricular tachycardia) or fibrillation (Table 3). For ischemic heart disease using non-ischemic heart disease as the referent, the hazard ratio was not statistically significant at 0.96 (95% CI 0.87, 1.07). The probability of an ICD discharge was greater in those who were prescribed an ACE inhibitor, an ARB, digoxin, or amiodarone. Similar risk factors were identified in a previous publication using this database which focused on ICD defibrillation in patients with a low ejection fraction who underwent ICD implantation for primary prevention [22].

**Table 3:** Hazard ratios for the occurrence of an appropriate ICD discharge among 10,320 patients in the ICD database stratified by baseline characteristics

Baseline characteristic	Hazard Ratio (95%CI)	***P***-value (2-sided)
Age (year)	1.01 (1.00,1.01)	0.0002
Male	1.60 (1.41,1.81)	< 0.0001
Smoke status		
Current cigarette smoker	1.15 (1.00,1.31)	0.049
Former smoker	1.14 (1.03,1.25)	0.009
Never smoker	Referent	
Primary heart disease		
Ischemic	0.96 (0.87, 1.07)	0.45
Non-Ischemic	Referent	
Implant indication		
Primary prevention	0.76 (0.68,0.85)	< 0.0001
Secondary prevention	1.57 (1.39, 1.76)	< 0.0001
Replacement	referent	
Atrial fibrillation	1.17 (1.06,1.28)	0.0011
Documented ventricular arrhythmia prior to ICD implant		
Tachycardia, fibrillation, cardiac arrest	2.06 (1.86,2.27)	< 0.0001
Non-sustained tachycardia	1.86 (1.63,2.13)	< 0.0001
None	Referent	
Left ventricular ejection fraction		
≤ 30%	1.00 (0.88,1.13)	1.00
31–40%	1.05 (0.90,1.21)	0.54
> 40%	referent	
Medications		
β-adrenoreceptor antagonist	1.06 (0.93,1.21)	0.37
ACE inhibitor or ARB^a^	1.20 (1.06,1.35)	0.0029
Spironolactone	1.07 (0.97,1.19)	0.17
Loop diuretics	1.07 (0.98,1.17)	0.14
Digoxin	1.17 (1.05,1.30)	0.0033
Amiodarone	1.27 (1.15,1.42)	< 0.0001

^a^Angiotensin-converting enzyme inhibitor or angiotensin II receptor blocker

### Case-crossover analysis of the associations between air pollution and ICD discharge

Analysis for this study was restricted to 1919 patients who experienced an appropriate discharge and could be linked with air quality and weather datasets. Case-crossover analysis did not demonstrate a significant association between an appropriate ICD discharge and any of the air pollutants for lags 0, 1, 2, 3 days and cumulative 4-day exposure when adjusted for 24 h mean temperature, relative humidity and barometric pressure (Table 4).

**Table 4:** Odds ratios (95% CI) for the association between the occurrence of an appropriate ICD discharge and an interquartile range increase in 24 h mean air pollutant concentrations (*n* = 1919). Results were adjusted for 24 h mean temperature, relative humidity and barometric pressure

Overall						Warm					Cold				
Air Pollutant	No lag	1- day lag	2-day lag	3-day lag	4-day mean	No lag	1- day lag	2- day lag	3-day lag	4-day mean	No lag	1-day lag	2-day lag	3-day lag	4-day mean
SO_2_, ppb	0.97 (0.91–1.03)	0.97 (0.91–1.03)	0.96 (0.91–1.02)	0.97 (0.91–1.03)	0.94 (0.85–1.03)	0.94 (0.86–1.02)	0.93 (0.85–1.02)	0.95 (0.87–1.03)	0.97 (0.90–1.06)	0.87 (0.76–0.99)	1.00 (0.91–1.09)	1.02 (0.93–1.11)	0.99 (0.90–1.08)	0.96 (0.88–1.05)	1.01 (0.89–1.15)
NO_2_, ppb	1.00 (0.89–1.12)	1.02 (0.91–1.14)	0.97 (0.87–1.09)	0.99 (0.89–1.11)	0.89 (0.75–1.05)	0.81 (0.65–1.00)	0.83 (0.68–1.03)	0.85 (0.69–1.04)	0.90 (0.74–1.10)	0.82 (0.60–1.13)	1.07 (0.94–1.23)	1.10 (0.96–1.26)	1.03 (0.90–1.17)	1.04 (0.91–1.18)	0.91 (0.74–1.11)
Ozone, ppb	0.97 (0.86–1.09)	0.98 (0.87–1.10)	1.02 (0.91–1.14)	1.03 (0.92–1.15)	1.02 (0.88–1.19)	1.02 (0.87–1.19)	1.00 (0.86–1.17)	1.03 (0.88–1.19)	1.05 (0.90–1.22)	0.97 (0.80–1.18)	0.92 (0.75–1.13)	0.94 (0.76–1.15)	1.02 (0.83–1.24)	1.05 (0.87–1.28)	1.14 (0.86–1.50)
PM_2.5,_ μg/m^3^ (adj)	0.99 (0.92–1.06)	0.99 (0.92–1.06)	1.00 (0.93–1.07)	0.99 (0.92–1.07)	0.97 (0.88–1.07)	0.94 (0.85–1.05)	0.96 (0.86–1.08)	0.99 (0.88–1.10)	1.01 (0.90–1.12)	0.94 (0.81–1.08)	1.02 (0.93–1.13)	1.00 (0.91–1.11)	1.01 (0.91–1.11)	0.99 (0.90–1.09)	0.99 (0.87–1.14)
AQHI	0.99 (0.90–1.08)	0.98 (0.90–1.08)	0.98 (0.89–1.07)	0.98 (0.90–1.08)	0.92 (0.82–1.03)	0.93 (0.82–1.06)	0.91 (0.80–1.04)	0.94 (0.82–1.06)	0.94 (0.83–1.08)	0.87 (0.75–1.02	1.05 (0.92–1.20)	1.06 (0.93–1.22)	1.02 (0.89–1.17)	1.05 (0.92–1.20)	0.96 (0.8–1.14)

We conducted models with restricted cubic spline terms for humidity, barometric pressure and temperature. The results using splines suggested that there was no significant non-linear relationship between weather and outcome. Apart from mean daily values, using splines for the one-hour maximum of the weather variables, and also using 8 h daily maximums for ozone did not significantly influence the results. Tests for an association between air pollution and ICD discharge were also stratified by each of the univariate predictors of ICD discharge found in Table 3. The 95% confidence intervals for all odds ratios included 1 for these many comparisons (results not shown) and therefore, were not statistically significant. To assess the sensitivity of the results to differences in geographic region we stratified the results by the five regions of Ontario and adjusted for weather variables (Table 5). For the period 2007–2012, the ranges in annual PM_2.5_ (μg/m^3^) for each region were: 6.21–7.85 for Eastern Ontario, 7.69–11.22 for Central Ontario, 7.76–9.92 Metropolitan Toronto, 8.35–10.15 for Southwestern Ontario, and 5.28–7.02 for Northern Ontario. No association between air pollution and ICD discharges were found in any of these regions.

**Table 5:** Odds ratios (95% CI) for the association between ICD discharge and an interquartile range increase in 24 h mean air pollutant concentrations stratified by the five regions of Ontario (*n* = 1919). Results were adjusted for 24 h mean temperature, relative humidity and barometric pressure

Air Pollutant	Region of Ontario				
	Eastern (***n*** = 426)	Central (***n*** = 738)	Metropolitan Toronto (***n*** = 349)	Southwestern (***n*** = 298)	Northern (***n*** = 108)
SO_2_, ppb	1.95 (0.89–4.28)	0.94 (0.83–1.07)	0.91 (0.70–1.20)	0.94 (0.80–1.12)	0.90 (0.45–1.77)
NO_2_, ppb	0.82 (0.46–1.45)	0.80 (0.62–1.02)	0.96 (0.70–1.30)	1.20 (0.75–1.93)	0.7 2 (0.14–3.74)
Ozone, ppb	0.85 (0.61–1.19)	1.10 (0.87–1.39)	1.10 (0.77–1.56)	0.98 (0.66–1.45)	1.04 (0.46–2.34)
PM_2.5_, μg/m^3^ (adj)	0.97 (0.75–1.26)	0.94 (0.81–1.10)	1.12 (0.92–1.36)	0.84 (0.65–1.10)	0.80 (0.40–1.62)
AQHI	0.87 (0.63–1.20)	0.88 (0.74–1.04)	0.93 (0.75–1.16)	1.11 (0.83–1.49)	0.64 (0.20–2.04)

## Discussion

The purpose of this study was to determine if day-to-day changes in air pollution in Ontario, Canada were associated with acute effects on potentially fatal cardiac arrhythmias. Potentially fatal cardiac arrhythmias, ventricular tachycardia and ventricular fibrillation were identified using appropriate ICD discharges (both defibrillation and overdrive pacing), an objective marker. Despite having a relatively large sample size we found no consistently positive association between air pollution and ICD discharges despite statistical testing of a large number of associations, lagging the data, and stratifying the data by multiple subject-related characteristics and by regions of the province which have different weather patterns and levels of air pollution. The one exception to finding no significant associations was the weakly negative association between SO_2_ and ICD discharge only in warm weather. We believe it is likely a chance association given that there is no biologically plausible reason that greater concentrations of SO_2_ should protect the heart. Multiple comparisons were made, and the confidence intervals for warm and cold season effects overlapped indicating that there was no significant interaction with season.

This study contributes to an area of uncertainty in the literature, whether or not potentially lethal cardiac arrhythmias can be triggered in patients with ICDs by low levels of ambient air pollution. Yang HJ et al. (2017) performed a systematic review and meta-analysis of studies which tested the association between air pollutants and ventricular arrhythmias in patients with ICDs [6]. Based on data from the seven studies included, the authors reported positive but non-significant associations. Pooled ORs (95% CI) were 1.03 (0.92, 1.17) for CO, 1.01 (0.97, 1.05) for PM_10_, 1.09 (0.95, 1.24) for SO_2_, 1.07 (0.95, 1.21) for PM_2.5_, 1.06 (0.98, 1.14) for NO_2_ and 1.00 (0.98, 1.01) for O_3_. Some of the individual studies had reported positive associations between ICD discharges and individual air pollutants. Kim et al. (2017) in a sample of 160 subjects in Korea found a positive association between air pollution and ventricular tachyarrhythmia, recorded by ICDs [23]. The authors commented that the ambient particulate exposure was rather unique, being influenced by dust storms from the Gobi desert [23]. Apart from the focus on ICD discharges, a review of 13 randomized controlled studies found no consistent evidence of a statistically significant association between air pollution from various sources and monitored cardiac rhythm in either healthy volunteers or those with stable cardiac disease [24].

The large number of patients in our study, almost 2000, was unique among studies of air quality and ICD discharges. The majority of studies in the systematic review of Yang et al. (2017) had less than 300 patients. Previous Canadian research in this area was done 16 years ago on patients with ICDs [13]. No significant association was found but the sample size was no more than 50 patients compared to the nearly 2000 in the current study. The large sample size allowed us to carry out subgroup analyses looking for the possibility of susceptible subgroups. Not all previous studies validated the ICD discharges to be in response to a ventricular arrhythmia. Dockery et al. pointed out that only 70% of discharges could be defined as appropriate as defined in the present study [25]. Many discharges may occur in response to supraventricular arrhythmias.

Most importantly, this study addresses the effect of relatively low concentrations of air pollution. Little is known about the effects of low concentrations of air pollution on ICD discharges. Though concentrations of NO_2_ and PM_2.5_ have been decreasing for decades in both the United States and Canada [10, 11], recent studies have found adverse effects of air pollution at concentrations within existing air quality standards [12]. Addressing this issue requires the availability of a patient group with monitored ICDs to be residing in an area of relatively low air pollution, and a large sample size necessary to have the power to detect small effects and allow subgroup analyses looking for particularly susceptible subgroups. To our knowledge, the present study has a combination of the largest patient population (although not the largest number of discharges), and the lowest observed concentrations of air pollution among the studies reported in the literature.

Our study contributes to the literature in several ways. Importantly, most studies were carried out in areas where air pollution was higher than in our study. PM_2.5_ ranged from 18 to 28 μg/m^3^ in a study done in Atlanta [26], 20 μg/m^3^ in a UK study [27], and between 19 and 27 μg/m^3^ in a study carried out over 20 years ago in Boston [28]. The mean PM_2.5_ in our study was 6.6 μg/m^3^.

Although adverse effects of air pollution at high concentrations have been recognized for decades, studying the health effects at lower levels is essential to determining what concentrations should be deemed safe or acceptable. This is especially important in developed countries where air quality has been improving over time. The air pollution concentrations observed in the present study were considered acceptable by American and Western European standards [29].

These findings are important for many reasons. A better understanding of the adverse health effects of air pollution exposure is important for its regulation in order to reduce morbidity and mortality. The general indication for an ICD implant is a high risk of experiencing a serious cardiac arrhythmia. Studying subgroups particularly susceptible to the adverse health effects of air pollution is important for several reasons. Air quality standards based on the observed effect size in the general population may be inadequate protection for all. Identifying the especially vulnerable would be a first step in focusing prevention strategies such as the Air Quality Health Index (AQHI). This unique index communicates to the public both a summary of the air quality and preventative advice which differs between those with and without an elevated risk due to chronic lung or heart disease and the general population [16]. The most susceptible may also derive the most benefit from a reduction in air pollution [30]. We found no evidence that air pollution increased the need for overdrive pacing or defibrillation to restore a more normal cardiac rhythm in this group at high risk of arrhythmias, even when stratified by other risk factors for arrhythmia.

One limitation of our study is the lack of personal exposure monitoring. Large epidemiologic studies rely on ambient air monitoring as an imperfect indicator of personal exposure. For case-crossover and time-series designs, the most common designs used to study acute effects of air pollution, we only need to assume that on days of higher air pollution, personal exposure on average is greater than on days where the ambient air pollution is lower. A systematic review of 18 studies found that the median correlation coefficient between personal and ambient concentrations of PM_2.5_ was 0.54 [31]. A study of 23 children reported a correlation coefficient of 0.41 between personal and outdoor ozone concentrations [32]. Day to day changes in personal exposure closely tracked changes in outdoor exposure which is particularly relevant to the current study where the change in pollution between case and control days, rather than the absolute level is the important independent variable. A study of 43 subjects in Boston, MA reported a correlation of 0.77 (95% CI 0.65, 0.89) between summertime ambient and personal PM_2.5_ [33].

There should not be any significant misclassification on the outcome since it was an objectively recorded electrical discharge and was subsequently determined to be an appropriate response. It would have been interesting to look at “inappropriate discharges” which may represent less severe arrhythmias but we did not have this information available. We imply that an appropriate ICD discharge is a good measure of a clinically important and potentially life threatening arrhythmia but we do not mean to equate a discharge with cardiac mortality. Unmeasured confounding by patient-related characteristics is unlikely. Each subject is compared to themselves so that these characteristics would be the same during case and control days. Any differences between case and control days in weather were statistically controlled. By controlling for day-of-the-week, we tried to minimize any differences in patients’ usual activity patterns between comparison days.

We looked at the association between air pollution and ICD discharges on the same day, and we also lagged the data to look for delayed effects. The study design addresses the effect of acute increases in pollution on ICD discharges. Acute effects of air pollution can be measured within hours in a controlled exposure setting [34]. Epidemiologic studies have detected acute effects of air pollution on cardiac and respiratory morbidity with short lags in the range we used. In a systematic review Mustafic et al. reported that increases in air pollution were associated with myocardial infarction with lags of 2 days for ozone, carbon monoxide, PM_10_, NO_2_, SO_2_, and PM_2.5_ [35]. We found no effect of daily air pollution on the same day, 1 day later, 2 days later and 3 days later. To address this issue further we calculated a 3-day cumulative exposure and included this in Table 4. Again, no significant effects were demonstrated. Using longer cumulative exposures would have averaged out daily peak exposures and reduced the differences between case and control exposure measures.

There are limitations to the generalizability of these results. This study addresses an important outcome in a unique population. Our findings should not be extrapolated to other cohorts with different characteristics. We have previously shown that air pollution was associated with a small increase in episodes of atrial fibrillation measured by Holter monitoring, and in a separate study also found that air pollution may influence the autonomic control of the heart [36, 37]. Our findings do not imply the absence of any adverse acute cardiac effects related to air pollution exposure. A review of biologic plausibility together with time-series studies argue strongly for a causal effect of short term increases in air pollution in cardiovascular death [38]. Postulated mechanisms include changes in vascular function, blood coagulability, oxidative stress and inflammation and autonomic nervous control of the heart [39]. Finally, our study of short term exposure does not address the long term adverse cardiac effects of chronic exposure to air pollution [40]. Relatively low air pollution concentrations could be considered a limitation to finding an effect, but the purpose of this study was to determine if there were effects discernable at current Canadian levels. Some older studies done at higher levels of exposure may have less relevance today because air pollution concentrations have decreased significantly over the past decade. The concentration of PM_2.5_ has decreased 44% between 1990 and 2015 in the U.S [41], and 25% between 2006 and 2015 in Ontario [42].

Strengths of this study include a well-characterized study group, a large number of person years of observation, objective measures of both exposure and outcome and the ability to assess the influence of a large number of patient characteristics on the strength of association between discharges and air pollution. We used lags to assess the possibility of time-dependent delays between exposure and response, and multiple stratifications looking for susceptible subgroups. Our findings of no association were consistent for each air pollutant tested despite stratification by the several predictive variables in Table 3 and stratification by region of Ontario. Concerning the power to detect a significant difference, Table 4 demonstrates that the confidence intervals are narrow, making it unlikely to miss an odds ratio larger than 1.2 for the overall results.

## Conclusions

In conclusion, daily increases in air pollution were not associated with increases in ICD discharges. The relatively low levels of air pollution in Ontario appear not to be an important risk factor for life threatening cardiac arrhythmias in this population.

## Data Availability

ICES which houses the ICD patient database does not allow its release even to Health Canada due to privacy regulations. Although Health Canada led the design and analysis of the study, the analysis had to be done in-house by ICES. The air pollution and weather data are publicly available.
